# Projecting the course of COVID-19 in Turkey: A probabilistic modeling approach

**DOI:** 10.3906/sag-2005-378

**Published:** 2021-02-26

**Authors:** Aybar C. ACAR, Ahmet Görkem ER, Hüseyin Cahit BURDUROĞLU, Seher Nur SÜLKÜ, Yeşim AYDIN SON, Levent AKIN, Serhat ÜNAL

**Affiliations:** 1 Department of Health Informatics, Graduate School of Informatics, Middle East Technical University, Ankara Turkey; 2 Department of Infectious Disease and Clinical Microbiology, Hacettepe University Faculty of Medicine, Ankara Turkey; 3 Department of Econometrics, Hacı Bayram Veli University, Ankara Turkey; 4 Department of Public Health, Hacettepe University Faculty of Medicine, Ankara Turkey; 5 Cancer Systems Biology Laboratory (KanSiL), Middle East Technical University, Ankara Turkey

**Keywords:** COVID-19, pandemic, epidemiology, Bayesian regression, Turkey

## Abstract

**Background/aim:**

The COVID-19 pandemic originated in Wuhan, China, in December 2019 and became one of the worst global health crises ever. While struggling with the unknown nature of this novel coronavirus, many researchers and groups attempted to project the progress of the pandemic using empirical or mechanistic models, each one having its drawbacks. The first confirmed cases were announced early in March, and since then, serious containment measures have taken place in Turkey.

**Materials and methods:**

Here, we present a different approach, a Bayesian negative binomial multilevel model with mixed effects, for the projection of the COVID-19 pandemic and we apply this model to the Turkish case. The model source code is available at https://github.com/kansil/covid-19. We predicted the confirmed daily cases and cumulative numbers from June 6th to June 26th with 80%, 95%, and 99% prediction intervals (PI).

**Results:**

Our projections showed that if we continued to comply with the measures and no drastic changes were seen in diagnosis or management protocols, the epidemic curve would tend to decrease in this time interval. Also, the predictive validity analysis suggests that the proposed model projections should have a PI around 95% for the first 12 days of the projections.

**Conclusion:**

We expect that drastic changes in the course of COVID-19 in Turkey will cause the model to suffer in predictive validity, and this can be used to monitor the epidemic. We hope that the discussion on these projections and the limitations of the epidemiological forecasting will be beneficial to the medical community, and policy makers.

## 1. Introduction

Coronaviruses are enveloped viruses with a positive-sense single-stranded RNA genome. Seasonal coronaviruses ( *HCoV-229E, HCoV-OC43, HCoV-NL63, HKU1-CoV* ) are some of the foremost causes of the common cold, and *SARS-CoV* and *MERS-CoV* are responsible for severe acute respiratory syndrome (SARS) and Middle East respiratory syndrome (MERS), respectively. These pathogens are the ones with the greatest impact on human health within the family *Coronaviridae* [1]. However, with the emergence of *SARS-CoV-2 —* the virus that causes COVID-191Republic of Turkey Ministry of Health (2020). COVID-19 Genel Bilgiler, Epidemiyoloji ve Tanı [online]. Website https://covid19bilgi.saglik.gov.tr/depo/rehberler/covid-19-rehberi/COVID-19_REHBERI_GENEL_BILGILER_EPIDEMIYOLOJI_VE_TANI.pdf [accessed 10 May 2020]. — in Wuhan, China, in December 2019, coronaviruses have become much more critical, and they attract the world’s attention without any doubt. Humankind has encountered one of the worst global health crises in the last 100 years [2]. Due to rapid dissemination, the World Health Organization declared a global pandemic on March 11th, 20202World Health Organization (2020). Coronavirus disease 2019 (COVID-19) Situation Report – 51 [online]. Website https://www.who.int/docs/default-source/coronaviruse/situation-reports/20200311-sitrep-51-covid-19.pdf [accessed 10 May 2020].. As of June 5th, 2020, there are 6,535,354 confirmed COVID-19 cases and 387,155 deaths worldwide3World Health Organization (2020). Coronavirus disease (COVID-19) Situation Report – 137 [online]. Website https://www.who.int/docs/default-source/sri-lanka-documents/20200605-covid-19-sitrep-137.pdf [accessed 05 June 2020]..

On March 11th, 2020, the Ministry of Health of the Republic of Turkey announced the country’s first confirmed COVID-19 case4Anadolu Ajansı (2020). Sağlık Bakanı Koca Türkiye’de ilk koronavirüs vakasının görüldüğünü açıkladı [online].Website https://www.aa.com.tr/tr/koronavirus/saglik-bakani-koca-turkiyede-ilk-koronavirus-vakasinin-goruldugunu-acikladi/1761466 [accessed 11 March 2020].. According to the official numbers, as of June 5th, 2020, there were 168,340 confirmed COVID-19 cases and 4,648 deaths in Turkey. Of the currently active cases, 592 patients were treated in intensive care units, and 269 of them were followed with invasive mechanical ventilation support5Republic of Turkey Ministry of Health (2020). Türkiye’deki Güncel Durum [online]. Website https://covid19.saglik.gov.tr/ [accessed 05 June 2020]..

From the date when the first confirmed patient was announced until today, a large number of social, political, economic, legal, military, religious, and cultural preventive measures were taken to slow the spread of the epidemic in Turkey; implementing curfews in metropolitan cities, establishing awareness of social distancing measures, national and international travel restrictions, closing of nonessential businesses, interrupting collectively religious ceremonies and postponement of summons, referral, and discharge procedures in military barracks are some examples of these measures. The full chronological list of the interventions is available upon request, as a resource for further studies. 

Immediately after the announcement of the COVID-19 epidemic in China, dissemination dynamics of the virus, and measures to prevent the spread, along with how the healthcare services should respond, were the urgent questions for researchers. Various modeling studies were initiated to tackle this task, such as the first modeling study on COVID-19 carried out by Wu et al., where they investigated the number of cases exported from Wuhan internationally to infer the number of infections in Wuhan from December 1st, 2019 to January 25th, 2020. They reported an estimated number of 75,815 individuals infected with *SARS-CoV-2* , which was much higher than the official numbers. Additionally, according to a remarkable finding, researchers stated that a 50% reduction in transmissibility would push down the viral reproductive number to about 1.3, which can significantly slow the epidemic and prevent a sharp peak during the first half of 2020 [3].

Researchers in the MRC Center for Global Infectious Diseases Analysis of the Imperial College of London have also been reporting their findings on the COVID-19 epidemic in China since January 2020. So far, they evaluated striking topics such as estimating the total number of patients, the efficiency of nonpharmaceutical interventions, the degree of online community involvement, the potential impact of the COVID-19 epidemic on other diseases such as HIV, tuberculosis, and malaria, and using mobility data to estimate transmission dynamics [4–8]. Meanwhile, in mid-March 2020, the Institute for Health Metrics and Evaluation (IHME) of the University of Washington published its empirical model [9]. The IHME started live forecasting at the state level for the USA and the national level for 17 selected countries6Institute for Health Metrics and Evaluation (2020). COVID-19 Projections 2020 [online].Website https://covid19.healthdata.org/united-states-of-america [accessed 10 May 2020].. They later expanded the number of countries projected to 50. The IHME started sharing their projections for Turkey COVID-19 recently, on May 15th, 2020. Likewise, the Robert Koch Institute (RKI) in Berlin published a new mechanistic model called SIR-X based on the confirmed cases for COVID-19 epidemic in China [10]. Their forecasting for 98 countries is also publicly available7Koch Institute (2020). Forecasts by Country [online]. Website http://rocs.hu-berlin.de/corona/docs/forecast/results_by_country/ [accessed 10 May 2020].. Later, Jianxhi Luo and their team from Singapore University of Technology and Design published foresight for 131 countries between April 18th, 2020, and May 11th, 2020 (white paper) based on a conventional mechanistic model known as SIR8Luo J, SUTD Data-Driven Innovation Lab (2020). Predictive Monitoring of COVID-19 [online]. Website https://web.archive.org/web/20200509191524/https://ddi.sutd.edu.sg/ [accessed 08 May 2020].. Several other real-time projections are also publicly shared online by different groups during the COVID-19 pandemic.

In this study, we have implemented a Bayesian negative binomial based multilevel mixed effects model inspired by IHME’s COVID-19 model for the projection of COVID-19 pandemic in Turkey from June 6th to June 26th. While presenting our projections here, we would like to open a discussion on the utility of these models for monitoring the dissemination and analysis of the effects of interventions during the COVID-19 pandemic in Turkey. 

## 2. Materials and methods

We model the progression of the epidemic in Turkey using a top-down empirical approach. The approach is similar to the COVID-19 model of the Institute of Health Metrics and Evaluation (IHME) of the University of Washington [9]. The COVID-19 projection of the IHME is a curve-fitting approach where the cumulative death curves in different states are fit with a logistic curve. Specifically, the scaled cumulative distribution curve of the Gaussian distribution. The base function is therefore of the form of (Eq. 1),

(1)p2(1+2π∫0α(t-β)e-t2dt)

where *p* is the scaling factor and determines the asymptotic limit, i.e., the ultimate number of total events (deaths or cases), α is the rate of increase in the number of events at the center of the epidemic wave, which in turn is β. One can thus think of the epidemic wave as a Gaussian kernel on the peak day (the day where the number of events is highest). The β parameter would then be the mean of this curve in terms of days (which is the unit of the independent variable *t* ), and α, proportional to the reciprocal of the standard deviation.

In the COVID-19 projection of the IHME, this curve is fit on the cumulative death data for each state in the U.S., Chinese provinces, and some European countries. They use the Gaussian kernel fit to the cumulative death rate of each location as a base kernel and generate 13 shifted versions of this base kernel. They then linearly combine them in a hierarchical generalized linear model (GLM) using the location (state/province/country), social distancing and lockdowns enforced for each location, and, more recently, cell phone mobility data as covariates. The COVID-19 projection of the IHME estimates the time-to-death from the day of infection, the case fatality rate, and thus the number of cases retrospectively, by working backward from the number of deaths in a particular location at a specific day to go back to the day those infections occurred. Projections of expected cases are calculated similarly by working backward from the predicted number of deaths.

As the number of confirmed cases is noisier and affected by the scale of testing and the testing policies in each region, the IHME uses the number of deaths. They contend that the number of deaths is a more reliable metric and thus have to perform this lagged estimation of cases. In the case of Turkey, the number of reported COVID-19 deaths are coupled to the number of confirmed cases. Specifically, for a death to be reported as a COVID-19 death, the patient has to be a confirmed case. So, in the case of Turkey, the number of confirmed cases is no more or no less reliable than the number of deaths. Therefore, we modeled directly on the confirmed case data and did not calculate the retrospective inference.

We also performed fitting differently than the IHME’s model. In addition to using a maximum likelihood curve fit, we modeled the uncertainty of our model using Bayesian regression. The IHME’s model calculates uncertainty by fitting the cumulative death numbers and generates confidence intervals from the parameter covariance and residuals of the MLE fit. One issue with this approach is that the cumulative numbers, by nature, are not independent. Each successive day’s sum is dependent on the previous days’ sums, as well as the underlying latent process. This dependency causes the projections of the IHME to underestimate uncertainty, which is also noticed by other researchers. One study has shown that the COVID-19 projections of the IHME (as published) have predictions that fall outside the 95% prediction interval in 49%–73% of the time [11]. The IHME team has updated their methodology subsequently to address these concerns, but those updates currently are not yet documented.

Instead of using cumulative numbers in calculating the predictive interval, we used the daily numbers of confirmed cases to prevent the serial dependency mentioned above. We used a Bayesian formulation with the generative model given in Eq. 2 below:

cl,t~NB(ml,t,r)

(2)ml,t=αlplNlπe-αl2(t-βl)2+ε

ε~N(0,σ)

Here, each day ( *t* ) of the epidemic for each location ( *l* ) is a draw from a negative binomial (NB) distribution with mean *m*_*l,t,*_ and reciprocal dispersion *r* . Namely, we model the number of cases each day and in each location as the count from a Poisson random variable with rate *m*_*l,t*_ . We allow overdispersion in this variable, hence we allowed for the choice of negative binomial distribution instead of Poisson. This mean count is *m*_*l,t*_ , which is derived from the base model in Equation 2. Here, *N*_*l*_ is the population, and *p*_*l*_ *, α*_*l*_ *, β*_*l*_ are the parameters for location *l* as discussed above. *ε* is the unbiased error term drawn from a normal distribution with 0 mean, parametrized by the covariance matrix of random effects. 

The model is essentially a 2-step negative binomial Bayesian regression where the posterior is parametrized by the expectation for the number of cases each day in each country, which is calculated by the output of the scaled Gaussian given in Equation 2.

The parameters for the above model have been estimated with the Hamiltonian Monte Carlo sampling, using the Stan (v2.19) [12] probabilistic programming platform under R (v3.6.1)9R Core Team (2018). R: A language and environment for statistical computing [online]. Website https://www.R-project.org/ [accessed 15 May 2020].. Default weakly informed priors provided by Stan were used for the parameters and the covariance matrix. The regression was done in the log space (using Stan’s neg_binomial_2_log parametrization), using 12 MCMC chains, with 2000 burn-in iterations, and 5000 sampling iterations in each chain.

## 3. Results

**Figure 1:**
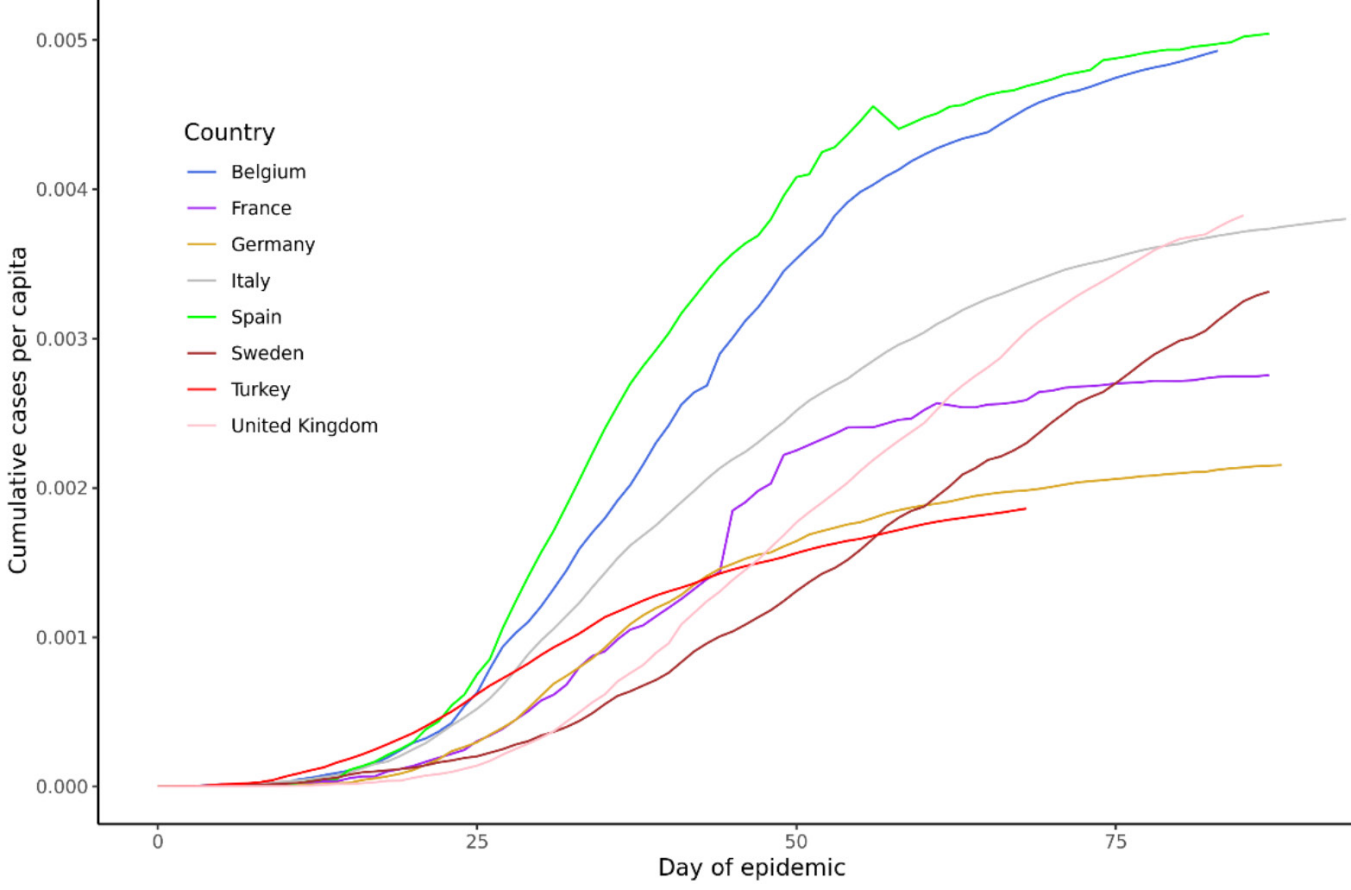
Progression of the epidemic in the countries used for analysis1John Hopkins University Coronavirus Resource Center. COVID-19 Map 2020 [2020-05-23]. Available from: https://coronavirus.jhu.edu/map.html.. The downward jump in the cumulative numbers for Spain originates from the original data source (JHU CSSE Coronavirus Tracker) when they readjusted the data to agree with the official Spanish Government figures.

We have fit the above model to the daily confirmed COVID-19 nationwide case numbers officially released by the Ministry of Health, Turkey until June 5th, 202010Republic of Turkey Ministry of Health (2020). Türkiye’deki Güncel Durum [online]. Website https://covid19.saglik.gov.tr/ [accessed 05 June 2020].11The Scientific and Technological Research Council of Turkey (2020). Türkiye’de Durum [online]. Website https://covid19.tubitak.gov.tr/turkiyede-durum [accessed 10 May 2020].. We used corresponding data for countries similar in epidemic progression to Turkey to estimate the random effects. The countries defined as the “locations” for the model are Turkey, Belgium, France, Germany, Italy, Spain, Sweden, and the United Kingdom. Day-zero for each country is when the number of cases surpassed 3 per 10 million population during the epidemic for that country. Figure 1 shows the 3-day moving averages of the case numbers in these countries shifted to match their day-zero and also scaled by their populations. Table 1 summarizes the key information for these countries. The ideal set of locations to use would have been different provinces in Turkey. Unfortunately, those data is not made publicly available. Therefore, we assume that this list of countries will allow us to estimate random effects relatively accurately. Our justification for this assumption is presented in the predictive validity section. 

**Table 1:** Countries Used in the Model: For each location, the day on which the number of cases surpassed 3 per 10 million population was taken as the day-zero of the epidemic in that location.

Country	Population as of May, 2020	Day zero of epidemic
Turkey	84.2M	March 17th
Belgium	11.6M	March 2nd
France	65.2M	February 27th
Germany	83.7M	February 26th
Italy	60.5M	February 21st
Spain	46.8M	February 27th
Sweden	10.1M	February 27th
United Kingdom	67.8M	February 29th

**Figure 2:**
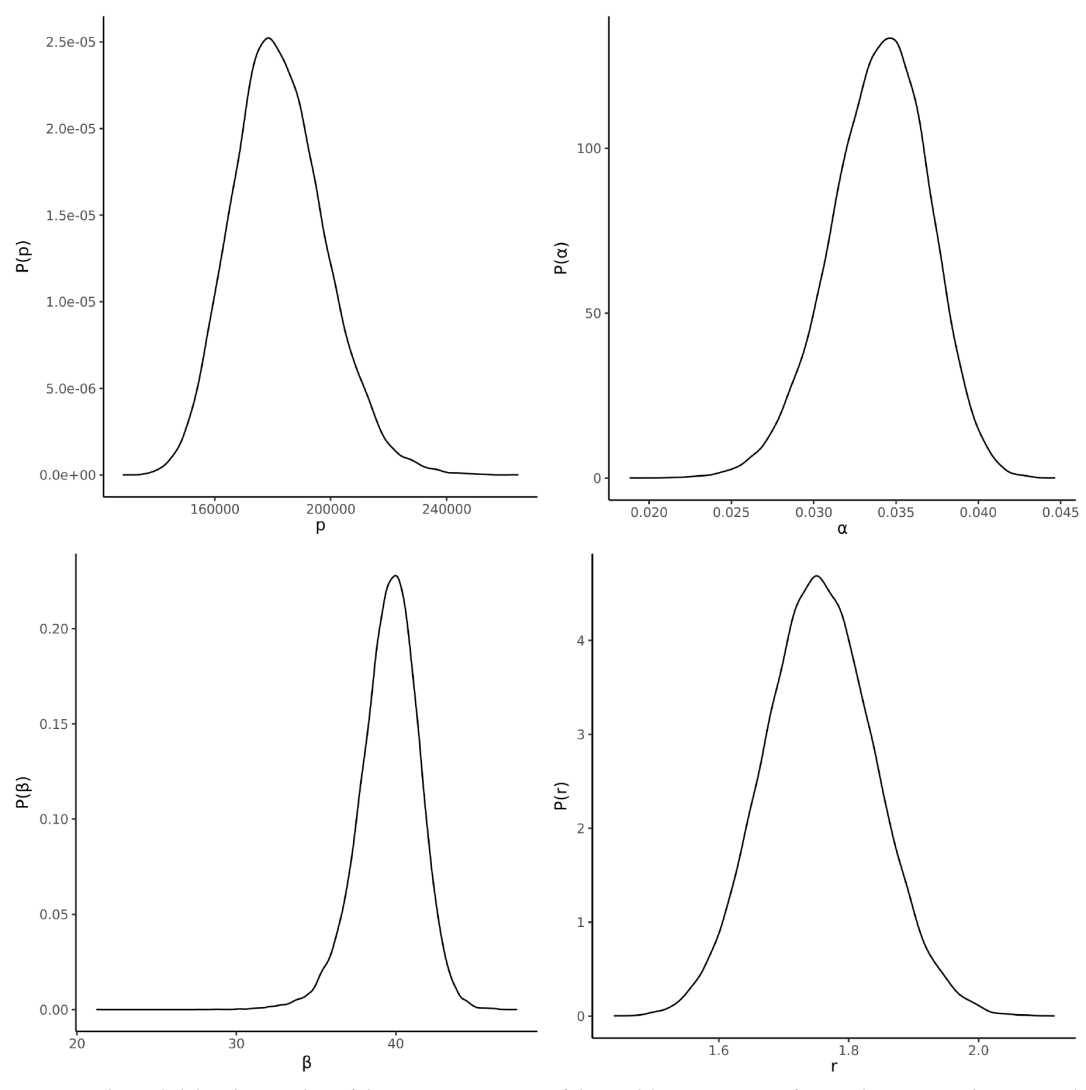
The probability density plots of the Bayesian estimates of the model parameters as of June 5th, 2020. r is the reciprocal dispersion parameter of the negative binomial. p is the asymptotic limit of the sigmoidal growth and is indicative of the total number of cases to be expected for this wave. α is the rate of growth at the steepest point of the curve, and β is the estimated center of the wave (the 40th day of the epidemic is April 26nd, 2020 for Turkey)

We ran the Hamiltonian Monte Carlo sampling on the official daily cases for Turkey from day-zero (March 17th, 2020) through to June 5th, 2020 (the present, as of this writing). The sampling converged very well with agreement among the chains, and there were no divergent traces. The description of the estimated posterior distribution parameters ( *r* , *p* , *α* , and *β* ) are as follows: *r* is the reciprocal dispersion parameter of the negative binomial; P is the asymptotic limit of the sigmoidal growth and is indicative of the total number of cases to be expected for this wave; α is the rate of growth at the steepest point of the curve, and β is the estimated center of the wave (the 40th day of the epidemic is April 26th, 2020 for Turkey) (Table 2 and Figure 2). 

**Table 2:** Distribution summaries of the model parameters as of June 5th, 2020: r is the reciprocal dispersion parameter of the negative binomial, p is the asymptotic limit of the sigmoidal growth and is indicative of the total number of cases to be expected for this wave. α is the rate of growth at the steepest point of the curve, and β is the estimated center of the wave (the 40th day of the epidemic is April 26nd, 2020 for Turkey).

Parameter	Mean	St.Dev	P25	P50	P75
r	1.732	0.083	1.674	1.730	1.787
p	183735	16170.6	172386	182843	193951
α	0.033	0.003	0.031	0.033	0.035
β	39.8	1.983	38.6	39.9	41.1

**Figure 3:**
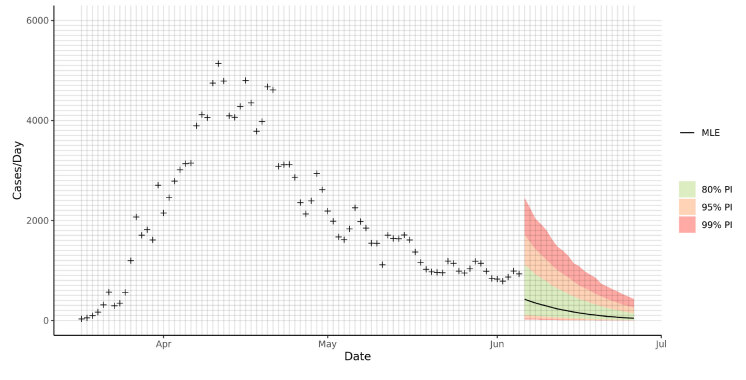
Predicted daily case numbers for June 6 – June 26. The green, orange, and red bands are the 80%, 95%, and 99% prediction intervals, respectively. The black line is the maximum likelihood point estimate (MLE). For example, on June 6 — our first prediction day — our maximum likelihood point estimate for the confirmed case number is 424, and 80%, 95%, and 99% prediction intervals are [114–1114], [46–1717], and [17–2454], respectively (see also Tables 3 and 4.

**Figure 4:**
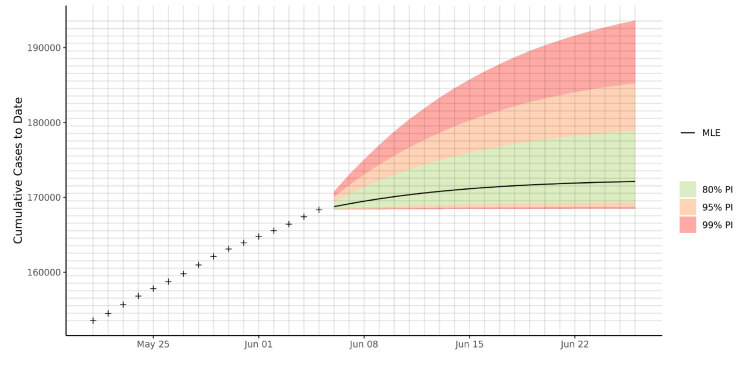
Predicted cumulative case numbers for June 6th – June 26th. The green, orange, and red bands are the 80%, 95%, and 99% prediction intervals, respectively. The black line is the maximum likelihood point estimate (MLE).

These posterior distribution parameters were estimated and used to sample the posterior predictives for the following 20 days after June 5th, 2020. The uncertainty (i.e., the prediction) intervals were found by taking the respective (80%, 95%, and 99%) quantiles of the posterior predictive sample. Figure 3 presents the prediction bands and the maximum likelihood point estimate for daily cases. Figure 4 is the cumulative form of the preceding figure and shows the predicted cumulative predictions until June 26th. The case number and cumulative number estimates of the model for the first, mid, and last day of the projection are listed as an example to show how our results should be interpreted. We expect [846–1717] confirmed cases and [168,386–170,057] cumulative cases for June 6th; [15–698] confirmed cases and [168,655–180,963] cumulative cases for June 16th; and [3–261] confirmed cases and [168,728–185,252] cumulative cases for June 26th, 2020 to be within the 95% PI (Figure 3–4 and Tables 3–4 ).

**Table 3:** Daily case number projections between June 6th and June 26th.

Date	Daily							
	60% PI		80% PI		95% PI		99% PI	
	Min	Max	Min	Max	Min	Max	Min	Max
6.06.2020	188	818	114	1114	46	1717	17	2454
7.06.2020	171	753	105	1025	43	1570	15	2249
8.06.2020	155	678	94	915	38	1425	14	2034
9.06.2020	139	621	84	854	33	1317	12	1927
10.06.2020	127	571	77	784	31	1213	11	1806
11.06.2020	114	510	68	698	28	1105	9	1625
12.06.2020	100	465	61	640	24	1007	8	1476
13.06.2020	90	422	54	588	22	931	8	1396
14.06.2020	82	381	49	528	18	863	7	1290
15.06.2020	72	342	43	480	17	777	5	1143
16.06.2020	65	307	39	428	15	698	5	1083
17.06.2020	58	276	35	392	13	646	4	983
18.06.2020	50	248	30	351	12	592	4	914
19.06.2020	44	223	26	316	10	529	3	853
20.06.2020	39	198	23	284	9	484	3	737
21.06.2020	34	175	20	253	7	434	2	683
22.06.2020	30	157	17	227	6	389	2	627
23.06.2020	25	139	15	204	5	355	1	578
24.06.2020	22	123	13	180	4	313	1	523
25.06.2020	19	108	11	161	4	286	1	479
26.06.2020	16	95	9	143	3	261	1	427

**Table 4:** Cumulative case number projections between June 6th and June 26th.

Date	Cumulative							
	60% PI		80% PI		95% PI		99% PI	
	Min	Max	Min	Max	Min	Max	Min	Max
6.06.2020	168528	169158	168454	169454	168386	170057	168357	170794
7.06.2020	168699	169911	168559	170479	168429	171627	168372	173043
8.06.2020	168854	170589	168653	171394	168467	173052	168386	175077
9.06.2020	168993	171210	168737	172248	168500	174369	168398	177004
10.06.2020	169120	171781	168814	173032	168531	175582	168409	178810
11.06.2020	169234	172291	168882	173730	168559	176687	168418	180435
12.06.2020	169334	172756	168943	174370	168583	177694	168426	181911
13.06.2020	169424	173178	168997	174958	168605	178625	168434	183307
14.06.2020	169506	173559	169046	175486	168623	179488	168441	184597
15.06.2020	169578	173901	169089	175966	168640	180265	168446	185740
16.06.2020	169643	174208	169128	176394	168655	180963	168451	186823
17.06.2020	169701	174484	169163	176786	168668	181609	168455	187806
18.06.2020	169751	174732	169193	177137	168680	182201	168459	188720
19.06.2020	169795	174955	169219	177453	168690	182730	168462	189573
20.06.2020	169834	175153	169242	177737	168699	183214	168465	190310
21.06.2020	169868	175328	169262	177990	168706	183648	168467	190993
22.06.2020	169898	175485	169279	178217	168712	184037	168469	191620
23.06.2020	169923	175624	169294	178421	168717	184392	168470	192198
24.06.2020	169945	175747	169307	178601	168721	184705	168471	192721
25.06.2020	169964	175855	169318	178762	168725	184991	168472	193200
26.06.2020	169980	175950	169327	178905	168728	185252	168473	193627

### 3.1. Evolution of the model parameters over time

**Figure 5:**
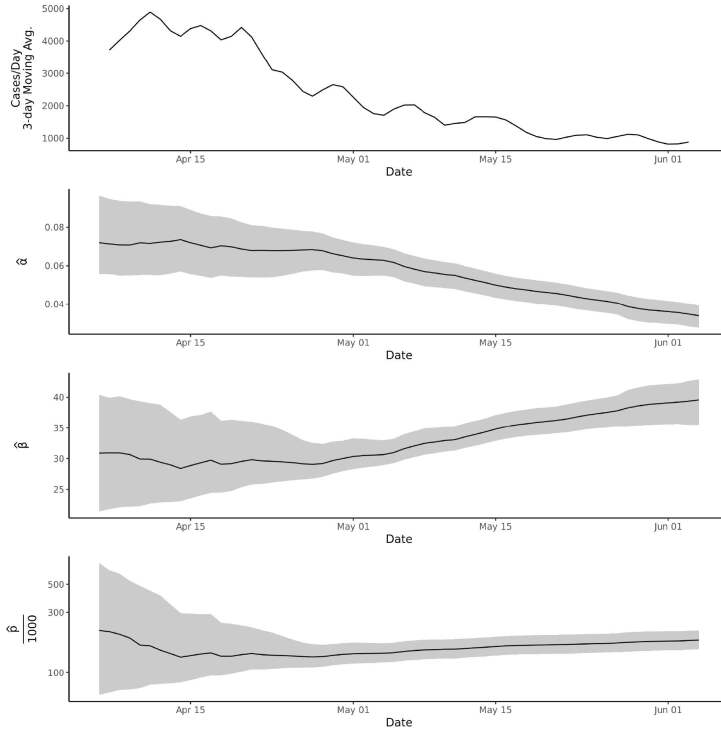
Evolution of the model parameter estimates over time, for regressions run each day from April 5 – June 5, 2020. The gray bands have the 95% confidence intervals. Note that the last plot (p) has a logarithmic y-axis.

We calculated the estimates of the parameters *p* , *α* , and *β* daily from April 5th, 2020, up to June 5th, 2020. For each day, the observations from the first day of the epidemic up to that day were fit, and the resulting trends plotted alongside a 3-day moving average of the daily case numbers. The resulting plot is shown in Figure 5. The noteworthy aspect of this analysis is that it demonstrates how much in flux the model parameters are until the day of maximum cases per day is reached. The parameter estimates stabilize after the peak, although there is still some drift.

### 3.2. Predictive validity

**Figure 6:**
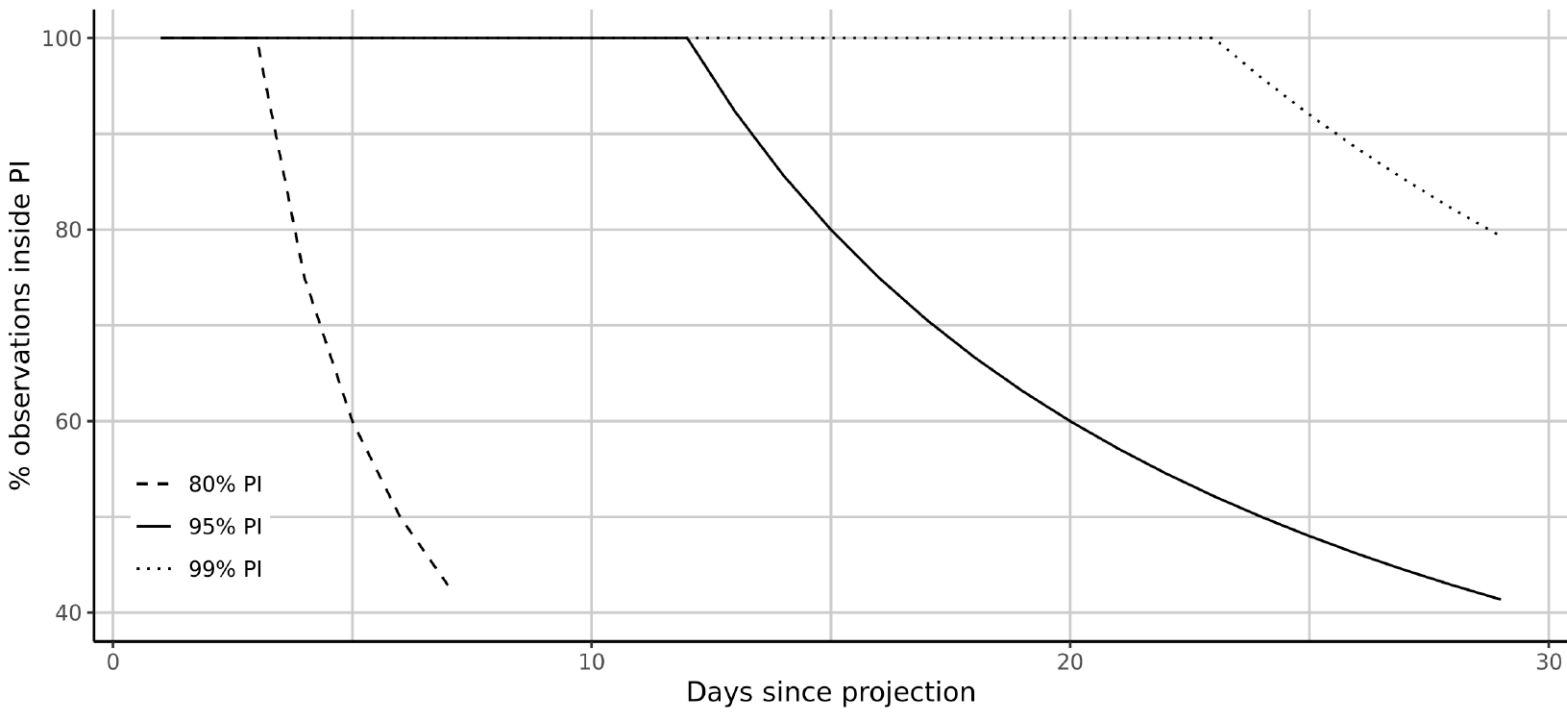
The predictive performance of the model for which the parameters were estimated with data up to May 5th, 2020. The plots show the percentage of future (May 6th – June 6th 2020) points that remain inside the different prediction intervals starting from the day after estimation up to a horizon of 30 days.

The predictive validity of the model is evaluated by rerunning the analysis only for the confirmed cases up to May 5th, 2020, holding the information for the last 30 days (between May 6th,2020 and June 5th, 2020) out of the analysis. The percentage of held out observations that remained inside the different prediction bands of the posterior predictive of the May 5 model was calculated. The results show that, nominally, the predictions are reliable within 10 days to 2 weeks into the future. The 95% prediction interval starts failing (i.e., the days that fall outside the interval become more than 5%) after 13 days. Likewise, the 99% prediction interval starts failing after 23 days (Figure 6). We, therefore, stipulate that our model would not be appropriate in making projecting for periods longer than about 20 days. The 20-day predictions as of June 5, 2020 are presented in Figures 3 and 4.

## 4. Discussion

Projecting the COVID-19 pandemic presents a challenge as it is a novel virus and even the dynamics of worldwide transmission are not known precisely. The basic reproduction number (R_0_) for COVID-19 is reported to vary from 2.2 to 5.7. While the main course of transmission is person-to-person, additional mechanisms of contact transmission with surfaces, objects, or even animals are also under investigation [13,14]12Australian Government - Department of Health (2020). Information for Clinicians: Frequently Asked Questions [online]. Website https://www.health.gov.au/sites/default/files/documents/2020/03/coronavirus-covid-19-information-for-clinicians.pdf [accessed 04 March 2020].. Moreover, the seasonal coronaviruses show strong and consistent seasonal variations. In various reports, hemisphere transitions and weather changes are thought to have significant effects on the course of the pandemic13Centers for Disease Control and Prevention (2020). How COVID-19 Spreads [online]. Website https://www.cdc.gov/coronavirus/2019-ncov/prevent-getting-sick/how-covid-spreads.html [accessed 10 May 2020]..

Developing an appropriate model to project COVID-19 requires comprehensive information [15–17]: Besides the transmission dynamics of COVID-19, individual, behavioral, and government-mandated containment measures also have significant effects on the routes of transmission [8,9]. For example, using masks decreases the infection rate by 70%–95%14Price A, Chu L, COVID-19 Evidence Service (2020). Addressing COVID-19 Face Mask Shortages [v1.1] [online]. Website https://stanfordmedicine.app.box.com/v/covid19-PPE-1-1 [accessed 10 May 2020].. After the announcement of the first COVID-19 case in Turkey, crucial interventions were set by the Turkish government like in many countries. Most of these interventions were implemented simultaneously or successively.

While struggling with these unclear conditions, many researchers and groups still try to produce mathematical models to forecast the future of the pandemic. RKI and SUTD have published their projections based on conventional epidemiological models. However, it is seen that epidemiological models applied to real-time modeling of epidemic or pandemic periods are very sensitive to the initial assumptions on multiple factors with significant variations. Modeling epidemics like the COVID-19 pandemic requires long-term analysis and high dimensional data. Hence, the central assumption of the SIR and SIR-X models, where all susceptibles were dropped from the transmission process by either infection or containment, is not valid, as no one will stay isolated entirely for extended periods. 

Giordano et al. published a study in which possible scenarios of the implementation of countermeasures were modelled, and they showed that restrictive social-distancing measures should be combined with widespread testing and contact tracing to control the pandemic [18]. For instance, if the lockdown is weakened in Italy, the number of patients may start to increase. Moreover, Ngonghala et al. showed in their modeling study that early termination of social-distancing measures might cause a new devastating wave in New York [19]. Prem et al. also highlighted the importance of physical distancing measures in their modeling study [20]. While these interventions are taking place, it will not be possible to analyze the course of the pandemic with the same assumptions, as the real-world circumstances are rapidly changing, and the projections of long-term case estimates can result in misleading results. Based on these arguments, RKI and SUTD have discontinued publicly publishing their worldwide projections based on the SIR-X and SIR models, respectively.

The COVID-19 projections of the UW/IHME, which inspired our model, assume a Gaussian distribution for the distribution of events (deaths or cases). However, as seen in Figure 3, the distribution of the confirmed cases in Turkey was not symmetrical as the Gaussian distribution assumes, and the number of new cases increased sharply. Still, it shows a gradual decrease causing the right skewness in the distribution. Therefore, in our generative model, we prefer to use negative binomial distribution, as stated in Equation 2. Even though we do not perform curve fitting for the distribution of the confirmed cases, we produce future projections of COVID-19 cases within reliable uncertainty bands. Several applications of negative binomial models are proposed, such as the assessment of the COVID-19 pandemic risk [21], demographic associations [22], or estimation of the distribution of the infection time [23]. Different from these studies, we have applied the Bayesian negative binomial multilevel model with mixed effects for COVID-19 case number modeling.

The conventional epidemiological models result in unrealistic overestimations, especially at the early stages of the spread. Similarly, the retrospective evaluation of our model showed a high flux in the model parameters before the day of maximum cases per day. Even though our projections showed a high variation in the early stages, as the spread continues with the accumulation of new data, it can project with lower flux for the estimates in 95% PI (Figure 5).

In the proposed model, we defined a 20-day forecast for Turkey with 95% PI. We anticipate that if we continue to comply with the measures and no drastic changes are seen in diagnosis or management protocols, the epidemic curve will tend to decrease in this time interval. During this period, we aim to investigate the epidemic curve dynamically by observing if the confirmed cases stay within the prediction intervals and monitor the course of the epidemic to give feedback on the effects of possible interventions to give insights into planners and policy-makers. An unexpected drift outside the PI bands will indicate the presence of a recent change in the course of COVID-19 spread in Turkey. These drifts in parameters are more likely to happen due to changes in the local interventions, such as business and curfew hours/days, public transportation, etc. 

There are several limitations to the study that need to be addressed. First, it should be noted that with the proposed methodology, we are not necessarily modeling reality but rather modeling the numbers. The model only reflects the real magnitude and the timeline of the pandemic to the extent the provided data are representative of the reality. Secondly, one can use many lagged covariates in the model: mobility information (from sources like Apple and Google), changes in the climate parameters, or lockdowns enforced, etc. We do not have precise information about these covariates, and about the lag between the exposure and the presentation of the symptoms. The inclusion of such lagged covariates is thus left as future work. 

Also, the model attempts to model random effects even though we missed data on the daily case numbers of individual provinces. We tried to overcome this limitation by using the data from a group of European countries to estimate the random effects. We cannot access data on mobility or preventive measures at the provincial level in detail. If these missing data are provided, the proposed model can also measure the efficacy of preventive measures independently. 

Lastly, the proposed model is ultimately a “single wave” model. If the current wave coincides with a new epidemic wave of significant size, both the accuracy and precision of the projections will drop dramatically. This limitation allows us to use the model as an early detection tool. If the model suddenly starts suffering significantly in predictive validity, this may indicate the beginning of a new wave. A multiwave model of the same form as the one we present here is possible: one in which the basis of the negative binomial is not a single Gaussian but a mixture of Gaussians. This type of extension to our approach and its ensuing research problems, like finding the minimum number of Gaussians needed to model a given set of observations, is also part of future work.

## 5. Conclusion

In this study, we propose a new methodology for the projection of COVID-19 pandemic inspired by the COVID-19 projections of the IHME. Intensive data requirements of epidemiological models and the fact that IHME’s COVID-19 projections tend to underestimate uncertainty led us to form our model. As a second wave is expected due to seasonal variations of coronaviruses, understanding the dynamics of the COVID-19 pandemic during the first wave through our model projections will be beneficial, and maybe also essential, for forecasting the efforts in the next stage, and the assessment of the response strategies. 

All models projecting COVID-19 provide estimations, and they should be utilized for assessing the effectiveness of various interventions rather than giving precise predictions. Currently, not only Turkey, but also many countries are progressively lifting their containment measures. The implementation of the reopening will mark the second phase of the pandemic, and monitoring based on the model projections is expected to be valuable to develop a well-defined strategy for the management of removing containment measures with a particular order and timeline. 
